# Clinical correlates of stress, immune and metabolic markers in major depression

**DOI:** 10.1192/j.eurpsy.2023.55

**Published:** 2023-07-19

**Authors:** V. Soria

**Affiliations:** ^1^Department of Psychiatry, Bellvitge University Hospital; ^2^Neurosciences-Psychiatry and Mental Health Group, Bellvitge Biomedical Research Institute (IDIBELL); ^3^Department of Clinical Sciences, University of Barcelona, Barcelona, Spain

## Abstract

**Abstract:**

The hormonal mediators of the stress response, such as glucocorticoids and catecholamines, have both protective and damaging effects on the body. In the short term, they are essential for adaptation, maintenance of homeostasis, and survival; but chronic exposure to stress or abnormalities in the modulation of the stress response can become maladaptive, leading to a broad range of physical and mental problems.

Allostatic load refers to the activation of physiological regulatory systems in response to stress and “the cost” of the effects of these systems on the body. Results from isolated biomarkers and allostatic load measures based on the stress response system (hypothalamic-pituitary-adrenal axis, autonomic nervous system and immuno-metabolic biomarkers) and its relationships with clinical outcomes, such as cognition, in a clinical sample of major depression patients will be presented. The usefulness and relevance in the clinical practice of those biomarkers and the allostatic load concept will be discussed. The integration of several biomarkers translating the biological and psychological impact of stress on depression development and its clinical trajectories could contribute toward understanding how to prevent and improve outcomes in major depression.

**Disclosure of Interest:**

V. Soria Grant / Research support from: Instituto de Salud Carlos III and the Bellvitge Biomedical Research Institute

